# Aetiology of Acute Lower Respiratory Infections among Children Under Five Years in Accra, Ghana

**DOI:** 10.3390/pathogens4010022

**Published:** 2015-01-26

**Authors:** Theophilus K. Adiku, Richard H. Asmah, Onike Rodrigues, Bamenla Goka, Evangeline Obodai, Andrew A. Adjei, Eric S. Donkor, George Armah

**Affiliations:** 1 Department of Microbiology, University of Ghana Medical School, Accra, Ghana; E-Mails: evaelli@gmail.com (E.O.); esampane-donkor@chs.ug.edu.gh (E.S.D.); 2 Department of Medical Laboratory Sciences, School of Allied Health Sciences, University of Ghana, Accra, Ghana; E-Mail: rhasmah@chs.edu.gh; 3 Department of Child Health, University of Ghana Medical School, Accra, Ghana; E-Mails: ponike@uhas.edu.gh (O.R.); bamenla@yahoo.co.uk (B.G.); 4 Department of Pathology, University of Ghana Medical School, Accra, Ghana; E-Mail: andrewanthonyadjei@yahoo.com; 5 Department of Electron Microscopy and Histopathology, Noguchi Memorial Institute for Medical Research, University of Ghana, Accra, Ghana; E-Mail: garmah@noguchi.ug.edu.gh

**Keywords:** acute respiratory infections, Respiratory Syncitial Virus, *Staphylococcus aureus*

## Abstract

The study aimed to investigate the aetiological agents and clinical presentations associated with acute lower respiratory infections (ALRI) among children under five years old at the Korle-Bu Teaching Hospital in Ghana. This was a cross-sectional study carried from February to December 2001. Nasopharyngeal aspirates and venous blood specimens obtained from 108 children with features suggestive of ALRI, were cultured and the isolated bacterial organisms were identified biochemically. Nasopharyngeal aspirates were also tested for Respiratory Syncitial Virus (RSV) antigen using a commercial kit (Becton Dickinson Directigen RSV test kit). A multiplex reverse transcription-PCR (RT-PCR) was also used to detect and characterize RSV using extracted RNA. Socio-demographic and clinical data were also obtained from the study subjects. Bronchopneumonia (55.5%), bronchiolitis (25%), lobar pneumonia (10.2), non-specific ALRI (4.6%), TB, bronchitis and respiratory distress (0.67%) were diagnosed. The prevalence of septicaemia was 10% and bacteria isolated were *Staphylococcus aureus*, *Streptococcus pneumoniae* and enteric bacteria, including *Salmonella* spp., *Enterobacter* spp and *Klebsiella* spp, were isolated. Out of the 108 cases, 18% tested positive for RSV, with two cases having RSV as the only aetiological pathogen detected. The subtyping analysis of RSV strains by a multiplex RT-PCR showed that subgroups A and B circulated in the season of analysis.

## 1. Introduction

In developing countries acute respiratory infections (ARI), diarrhoeal diseases, malaria and malnutrition are major causes of death among young children [1]. It is estimated that 18% of all deaths in children less than five years old are attributable to ARI as an underlying or contributory cause, thus indicating ARI as the commonest cause of the deaths [2]. Infections of the lower respiratory tract are known to be important causes of morbidity as well as a leading reason of hospitalization in children in Africa [3].

Several agents have aetiologically been associated with severe ALRI (acute lower respiratory infections). These include viral agents, such as RSV, adenoviruses, influenza virus, human metapneumovirus and para-influenza virus [4,5]. A wide range of bacterial agents are also implicated in ARLI and include *Streptococcus* *pneumoniae*, *Staphylococcus aureus*, *Haemophilus influenzae*, *Moraxella catarrhalis*, *Escherichia coli*, *Salmonella* *species Mycoplasma pneumoniae,* and *Chlamydia pneumoniae* [6,7,8]. Respiratory syncytial virus (RSV) has been identified as causing proportionately more ALRI than the others particularly during the first year of life [9,10]. RSV is responsible for approximately 85% of cases of bronchiolitis and 20% of cases of childhood pneumonia [9,10]. A recent systematic review showed that RSV is responsible for 66,000 to 199,000 deaths annually among children less than five years old [11]. Vigorous initiatives are hence being developed for the prevention or reduction of RSV disease worldwide. These among others include the development of vaccines for both childhood and maternal immunization.

Whilst ALRI disease has been well documented in industrialized countries, the same cannot be said of developing countries. From the few population based estimates of the incidence of RSV disease in the developing world, the rate has been estimated between 540 to 1000 episodes per 1000 child years with peak infection in infants less than one year old [12]. In Africa, there are few reports on ALRI from Zambia [13], Ethiopia [14], Gambia [15], South Africa [16] and Kenya [17], and there is therefore paucity of data on RSV serotypes circulating on the continent. In Ghana, the first study on ALRI was in 1991 where clinical diagnosis was done with no laboratory-based identification and confirmation of the etiological agents [18]. In the present study, we investigated the aetiology of ALRI in children under five years of age in the Greater Accra region of Ghana.

## 2. Results

**Figure 1:**
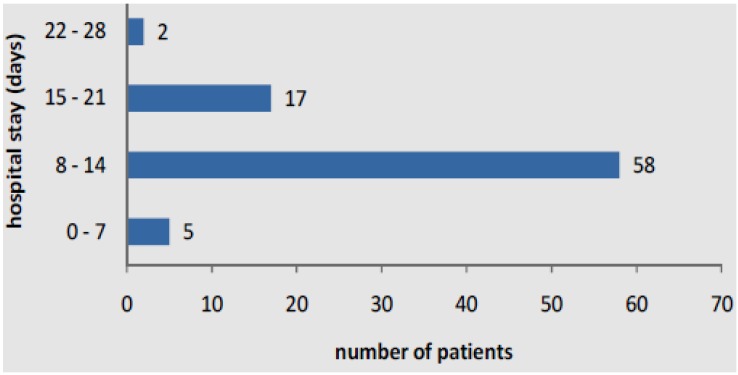
Duration of stay in hospital of children with acute lower respiratory infections.

The 108 children sampled in the study comprised 53 males and 55 females; their age range was 0–47 months, with majority of them (78/108) falling in the age group of 0–11 months (Table 1). About 8.3% had experienced similar illness in the past and 54.6% received antibiotics before admission (Table 2). Majority of the study subjects (54%) were hospitalized for 8–14 days (Figure 1).

**Table 1:** Distribution of study population by age, sex, and proportion hospitalized.

Age	Males	Females	Proportion Hospitalized
0–2 months	14	15	26.9
3–5 months	12	13	23.1
6–11 months	12	12	22.2
12–23 months	9	7	14.8
24–35 months	6	5	10.2
36–47 months	0	3	2.8
48–59 months	0	0	0

**Table 2:** Demographic data of children with acute lower respiratory infections.

Factor	Details	Percentage
Past illness and immunity	Similar illness in the past	8.3
	Exclusive breast feeding up to 6 months	86.1
Subject’s association with other children	Siblings with same illness	17.9
	Child attends day-care center	9.3
	Child sleeps alone	4.7
Subject’s household factor	Single parents	7.5
	Median (range) no. of people in the room	5 (2–9)
	Passive smoking in the household	20.6
Self-medication (prior to admission)	Antibiotics	54.6
	Antimalarials	56.1
	Cough mixture	41.7
	Herbal medicine	8.3
	Paracetamol	78.9

**Figure 2:**
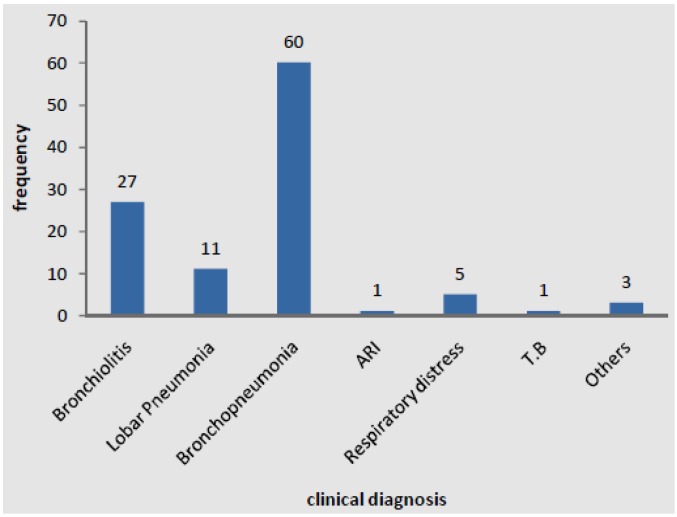
Clinical diagnosis of children with acute lower respiratory infections.

**Figure 3:**
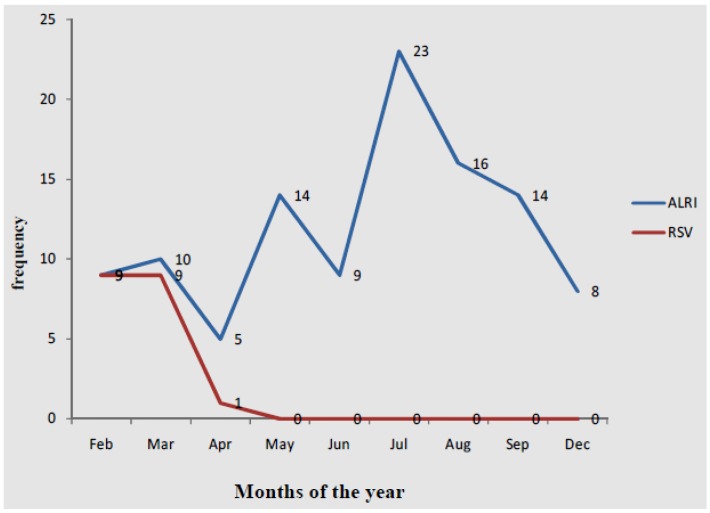
Monthly distribution of cases of acute lower respiratory infections and Respiratory Syncytial Virus.

Overall, eight fatal outcomes were reported and were associated with several clinical diagnosis including meningitis, septicaemia, HIV infection and gastroenteritis. The overall prevalence of RSV infection amongst ALRI patients was 18%. The most frequent symptoms were cough (95.4%), difficulty in breathing (89.8%), fever (83.3%) and diarrhea (25.9%) (Table 3). The most frequent causes of hospitalization of RSV patients were bronchopneumonia, bronchiolitis, pneumonia and respiratory distress (Figure 2). Generally, there were no significant differences in clinical characteristics between children who had RSV infection and other those who had other ALRI. The monthly distribution of ALRI and RSV cases are reported in Figure 3. ALRI cases occurred non-uniformly throughout the 11 months study (February to December) and peaked in July at 23 cases. RSV cases peaked in February and March (nine cases) after which it declined with no reported cases from May.

**Figure 4:**
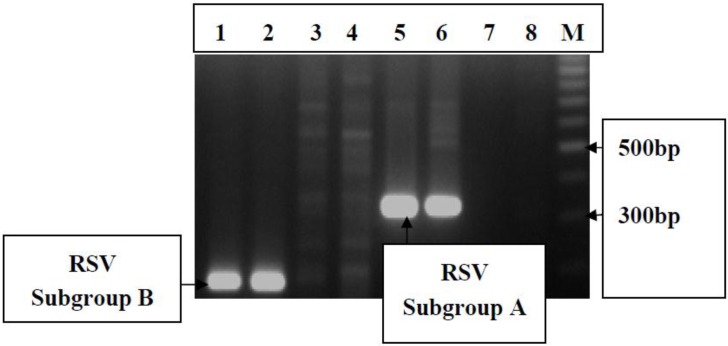
Electrophoregram showing type specific identification of Respiratory Syncitial Virus (RSV) groups from nasopharyngeal aspirates. Lane M is a 100 bp DNA molecular weight marker. Lanes 1 and 2, RSV group B (183 bp); lanes 5 and 6, RSV group A (334 bp); and lane 7 negative control using cDNA from rotavirus positive sample and lane 8, negative control.

Based on bacterial culture results (Table 4), 10% of the children had evidence of septicemia and the implicated bacterial agents were *Staphylococcus aureus*, *Streptococcus pneumoniae* and a number of enteric bacteria including *Salmonella* spp., *Enterobacter* spp and *Klebsiella* spp. There were six cases (5.6%) of septicaemia and ALRI/RSV infection. Culture of throat swabs and nasopharyngeal specimens yielded a wide range of organisms (Table 4), most of which are consistent with their occurrence as normal flora at these anatomical sites. The most prevalent bacterial organism isolated from the throat was *Staphylococcus aureus* (8%), followed by *Streptococcus pyogenes* (5%), *viridians streptococci* (4%) and *Candida spp*. (4%). The most prevalent bacteria isolated from the nasopharynx was *Staphylococcus aureus* (16%) but enteric organisms including *Enterobacter* spp (6%), *Klebsiella* spp. (4%) and *Escherichia coli* (4%) were also common at this anatomical site (Table 4). Nineteen samples (18.4%) were positive on the RSV Becton-Dickenson Rapid test. Viral RNA was extracted from eight of the positive samples and multiplex PCR analysis showed that two samples were RSV group A and two were RSV group B (Figure 4).

**Table 3:** Clinical presentation of children with acute lower respiratory infections.

Indication	Details	% of patients (n = 105)
Symptoms		
	Cough	95.4
	Difficulty in breathing	89.8
	Wheezing	15
	Stridor	0.9
	Nasal discharge	62.0
	Difficulty in feeling	53.3
	Fever	83.3
	Diarrhoae	25.0
Mental disposition		
	Normal	68.2
	Irritable	20.6
	Lethargic/drowsy	9.3
	Unconscious	1.9
Chest signs		
	Chest indraw	44.9
	Bronchial breathing	14.2
	Ronchi	17
	Crepitations	69.8
	Chest splinting	0
	Grunting	12.3
Therapy options		
	Bronchodilators	10.3
	Oxygen	51.4
	Antibiotics	96.3

**Table 4:** Bacteria isolated from blood, throat swabs and nasopharyngeal specimens.

Bacteria	Specimen		
	Blood	Throat sp	Naso sp
*Staphylococcus aureus*	4	8	16
*Streptococcus pneumoniae*	1	1	3
*Salmonella spp*	3	0	0
*Enterobacter spp*	1	1	6
*Klebsiella spp*	1	2	4
*Streptococcus pyogenes*	0	5	0
*Moraxella catarrhalis*	0	1	3
*Viridans streptococci*	0	4	2
*Pseudomonas aeuroginosa*	0	0	3
*Escherichia coli*	0	0	4
*Proteus spp.*	0	0	1

## 3. Discussion

We found that prevalence of ALRIs due to RSV infection in children observed in the present study was 18%, which is relatively higher than the prevalence rates reported in several developing countries such as The Gambia, Zambia and Eithopia [13,14,15]. It is interesting to note that while ALRI cases appeared to occur throughout the year, RSV cases were confined to the early part of the year. This highlights the importance and role of other aetiological agents of ALRI among the study subjects, apart from RSV. Though we isolated a wide range of bacterial agents from the study subjects, the spectrum of ALRI aetiology may be more extensive, including parasites and viruses that we did not screen for. Bronchopneumonia and bronchiolitis were the most severe complications associated with ALRI infections, which concurs with previous studies [19,20]. The symptoms of ALRI infection are similar to those of several bacterial infections and also malaria. This may explain why prior to visiting the study hospital, majority of the study patients had been on antibiotics or antimalarial agents. Discussions with physicians who took care of the patients also showed that all RSV infections received antibiotics while on admission. This depicts RSV infection as a leading cause of prescription for antibiotics and antibiotic misuse.

All but eight patients recovered resulting in a 7.4% mortality rate, which is higher than rates reported in several countries [21]. The high rates of morbidity of RSV infection observed in the current study can have a large unfavorable impact on public health. For example, majority of the patients spent more than a week on hospital admission, which can be a major burden on the hospital facilities, such as bed turnover, staff, and high expenditure on maintaining patients per day. This can also be stressful on the families involved, as parents have to absent themselves from work and home, placing strain on the financial resources of the family. The high case fatality observed in this study requires serious attention in the light of achievement of Millennium Development Goal (MDG) 4, which is to reduce child mortality by two-thirds.

*S. aureus*, which was the most common bacterial organism in the nasopharyngeal aspirate samples, is known to occur as a normal flora in the nasopharynx of most healthy children. Consequently, its occurrence in septicemia among the children may be indicative of endogenous sources of infection. However, molecular studies are needed to confirm this. Our data shows that *S. aureus* may be an important cause of septicaemia among children in Ghana. The absence of organisms like *Haemophilus influenzae* and *Neisseria meningitidis* in nasopharyngeal aspirates is surprising as these organisms are normal residents in the nasopharynx. *Haemophilus influenzae* and *Neisseria meningitides* are fastidious organisms, and the culture method used may not have been sensitive enough to detect them.

The multiplex-PCR assay was able to detect and confirmed the presence of RSV and thus useful in assessing the contribution of RSV to the overall burden of respiratory illness in the community and can be of use in diagnostic settings where subtype information might be sought. Both group A and B RSV were found to co-circulate during the study period. The results from this study will enhance the clinical management of ALRI as well as provide information for evaluating any future vaccines; especially for RSV.

There are a few limitations of the study. Firstly, our ALRI surveillance did not cover a full year and cases in January may have been missed resulting in an under-estimation of RSV prevalence. Secondly, the small volume of blood used for the cultures may have resulted in low sensitivity of detection of bacterial organisms in the blood. Additionally, nasopharyngeal aspirates may not be very useful for diagnosis of ALRI due to bacteria and isolates from blood does not necessarily imply causation of ALRI. Thirdly, only the RSV positive rapid antigen tests were tested by PCR for subgrouping due to scarcity of resources. The PCR test in general is more sensitive than the rapid antigen test, and thus additional cases could have been identified if the PCR method was used to evaluate all the samples. Our data on RSV subgrouping is limited and we recommend further studies using a larger sample size to determine RSV subgroups in the country.

## 4. Methods

The methodology for the study was based on the WHO generic protocol to examine the incidence of lower respiratory infection due to RSV in children less than 5 years old [22]. Standard protocols and control strains were employed in the laboratory investigations.

### 4.1. Study Site and Population

This was a cross-sectional study carried out at the Department of Child Health at the Korle-Bu Teaching Hospital located in Accra, Ghana. The Korle-Bu Teaching Hospital is the major referral Hospital in Ghana with a daily outpatient attendance of more than 1000. Prospectively, one hundred and eight (108) children were recruited consecutively into this study from February to December 2001. This included all children under 5 years old with ALRI who sought medical assistance at the department of Child Health. All the ALRI cases in the study were community acquired and there were no secondary referrals from other hospitals. All the study children had received the *Haemophilus influenzae* Type b vaccine but none of them had received pneumococcal vaccination. The bio-data of the study subjects recruited into the study were obtained by administering a questionnaire. Risk factors, such as malnutrition, overcrowding, and passive smoking, were recorded. Known asthmatics and children with abnormal cardiovascular systems were not recruited in the study. ALRI was defined according to the age of the subject as follows. For children <2 months, breathing rate of 60 per minute or severe chest in-drawing or stridor or wheezing or apnea. For children 2–11 months, breathing rate of 50 per minute or chest in-drawing or stridor or wheezing or apnea. Finally, for children 12–59 months, breathing rate of 40 per minute or chest in-drawing or stridor or wheezing or apnea. The admitting physician managed all children recruited into the study according to the standard departmental protocol for ALRI.

The study was approved by the Ethical and Protocol Review Committee of the University of Ghana Medical School and written informed consent of the parents or the caregiver of children was obtained before sample collection.

### 4.2. Sample Collection

Nasopharyngeal aspirates, throat swabs and blood samples were collected from the study children after they were diagnosed of ALRI. Nasopharyngeal aspirates and throat swabs were collected from all the children while venous blood samples were collected from one hundred and four of them who were fit for bleeding. Nasopharyngeal aspirates were obtained using the sterile single-use Argyle^®^ Deele Suction catheter with mucus trap kit. Initially, nasopharyngeal aspirates were collected by aspiration through a catheter and then washed down into the attached tube with 2 mL sterile saline solution as transport medium. The various types of samples collected were transported on ice to the Microbiology Laboratory of the University of Ghana Medical School located in Accra, the capital city of Ghana. A rapid diagnostic test to detect RSV was carried out on nasopharyngeal aspirates using the Becton-Dickenson RSV Kit (recommended by WHO). Nasopharyngeal aspirates, throat swabs and blood samples were cultured for isolation and identification of bacteria. Details of the laboratory investigation are described as follows:

### 4.3. Culture and Identification of Bacterial Isolates

Blood samples were inoculated into tryptone soy and thioglycolate broths, and then subcultured onto sheep blood agar and chocolate agar [23,24]. Throat swab and nasopharyngeal specimens were cultured on sheep blood agar and enriched chocolate agar [23,24]. Bacterial isolates obtained from the cultures were identified based on colonial morphology Gram staining and a battery of biochemical reactions [19,20,21,22]. *Streptococcus pneumoniae* and viridian streptococci were differentiated by Optochin sensitivity [19]. *Staphylococcus aureus* was identified by Coagulase test [23]. *Haemophilus* spp. and *Moraxella* *catarrhalis* were identified using an *in vitro* diagnostic system (Becton Dickinson, Franklin Lakes, NJ, USA) according to the manufacturer’s recommendations. *Streptococcus pyogenes* was identified by Bacitracin susceptibility. Gram negative rods were identified by Analytical Profile Index.

### 4.4. RSV Becton-Dickenson Rapid Test

RSV was identified from fresh aspirates using the WHO recommended Becton-Dickenson Rapid RSV diagnostic kit according to the manufacturer’s recommendations. All positive samples were analyzed by PCR for confirmation of antigen positivity as well as the differentiation of serotypes.

### 4.5. RSV Viral RNA Extraction, Reverse Transcription (RT) Reaction and Multiplex PCR Analysis

Viral RNA was extracted from RSV using RNaid extraction kit (Q BIO gene, UK). The extraction process was done following the manufacturer’s instruction. The RNA extracted samples were stored at −20 °C. The purified ssRNA was reversed transcribed as described by Stockton *et al.* [25] with slight modifications. Briefly, 1 µL of hexamer random primers (20 mU; PdN6; Pharmacia Biotech) was added to 22.2 µL ssRNA template. To this was added 17.8 µL of master mix. The master mix comprised 3.5 µL 5X Go buffer (Promega, USA), 10U avian myeloblastosis (AMV) reverse transcriptase (Promega, USA), 1 µL 10 mM, dTTP, 1 µL 10 mM dATP, 1 µL 10 mM dCTP, 1 µL 10 mM dGTP and 8.1 µL RNase free water. The mixture was incubated at room temperature for 10 min at 42 °C in a Perkin Elmer GenAmp 2700 PCR Machine (Applied Biosystems, Middletown, CT, USA) for 45 min and 95 °C for 5 min to inactivate the AMV enzyme. Two negative controls were set up using 22.2 µL of RNA sample known to be positive for rotavirus and nuclease free water. The cDNA products generated was stored at −20 °C.

The N and P regions of the RSV genome were amplified using a forward primer (RSV AB F) and a reverse primer (RSV AB R). The primers described by Stockton *et al.*, [25] were used in the PCR analysis. Briefly, the slightly modified PCR reaction mix of 50 µL contained 5X Go PCR buffer (Promega, Madison, WI, USA), 200 µM of each of the 4 oligonucleotide triphospates (dNTPs) (Promega, USA), 5 pmols of each primer, 1.6 U RNasin (Promega, USA) and 1.5 units of Taq Polymerase enzyme (Promega, USA). Ten microliters of cDNA product from the RT reaction was used as template for the amplification reaction. Nuclease free water was used to make up the volume to 50 µL. The reaction mix was spun down for 30 s at 14,000 rpm and the amplification was carried out using a Primus 25 Advance PCR machine (PEQLAB, GmbH, Erlangen, Germany). The cycling parameters for the 1st round PCR with the primers were as follows: 94 °C for 2 min, followed by 35 cycles of 94 °C for 1 min, 50 °C for 1 min, 72 °C for 1 min. For each reaction, a positive control (RSV positive sample) and a negative control (no DNA template added) were run. In the second round multiplex PCR, 1 µL of first round PCR product, 25 pmols of forward and reverse primers for subtyping were used. The other parameters above remained the same. The PCR cycling parameters were as follows: 94 °C for 2 min, followed by 35 cycles of 94 °C for min, 60 °C for 30 s, 72 °C for 1 min. Primers for the identification of RSV subtypes A and B were used. On completion of the PCR, the products were electrophoresed on a 2% agarose gel and stained with 0.5 µg/mL ethidium bromide to detect the presence of amplified DNA fragments. Ten microliters of each sample was added to 2 µL of bromophenol (5X) gel loading dye for the electrophoresis. Hundred base pair DNA molecular weight marker (Promega, USA) was run alongside the PCR products. The gel was prepared and electrophoresed in 1X TAE buffer using a mini gel system at 100 volts for one hour and the gel photographed over a UV transilluminator.

### 4.6. Analysis of Data

All data were double entered and stored on a microcomputer. Analysis of the generated data was carried out using EPI Info Version 16 software (CDC, Atlanta, GA, USA). The strategies taken to analyze the data involved descriptive statistics, including geometric means, frequencies, ranges and prevalence rates of the study variables. Significant differences, associations and interrelationships of the variables were also assessed at a level of *p* < 0.05.

## 5. Conclusions

The study concludes that ALRI has an annual distribution in Accra, and peaks around the middle of the year (July). RSV accounts for a significant proportion of the ALRI and RSV cases appear to be highly seasonal, occurring in the early part of the year. Apart from RSV, several other microbial agents are involved in ALRI in Accra, and a more targeted epidemiological study of these causative organisms is required.
